# Clinical Outcomes of Modified Manual Deep Anterior Lamellar Keratoplasty for Eyes with Previous Radial Keratotomy

**DOI:** 10.3390/jcm13175250

**Published:** 2024-09-05

**Authors:** Francesco Aiello, Giulio Pocobelli, Alfonso Iovieno, Chiara Komaiha, Carlo Nucci, Augusto Pocobelli

**Affiliations:** 1 Ophthalmology Unit, Department of Experimental Medicine, University of Rome “Tor Vergata”, 00133 Rome, Italy; 2 Moorfields Eye Hospital NHS Foundation Trust, London EC1V 2PD, UK; 3 Department of Ophthalmology and Visual Sciences, University of British Columbia, Vancouver, BC V5Z 3N9, Canada; 4 Providence Health Care, Vancouver, BC V5Z 3N9, Canada; 5 Ophthalmology Unit—Eye Bank of Rome, San Giovanni Addolorata Hospital, 00184 Rome, Italy

**Keywords:** deep anterior lamellar keratoplasty, big-bubble technique, manual dissection, penetrating keratoplasty, radial keratotomy

## Abstract

**Background**: The aim of this study was to evaluate the intraoperative complications and visual outcomes of manual deep anterior lamellar keratoplasty (mDALK) in patients who underwent previous radial keratotomy (RK) for myopia. **Methods**: The notes of patients who underwent mDALK after RK at three different hospitals—San Giovanni Addolorata Hospital (Rome, Italy), Mount Saint Joseph Hospital (Vancouver, Canada), and Tor Vergata University Hospital (Rome, Italy)—were retrospectively reviewed. We analyzed the manual dissection success rate and conversion to penetrating keratoplasty (PK), the residual recipient stromal thickness, the postoperative corrected distance visual acuity (CDVA), postoperative refraction, and topographic astigmatism. **Results**: Thirteen eyes of eleven patients were included in the analysis (male 7/11, 63.6%). Preoperatively, mean topographic astigmatism was 5.4 ± 3.5 D (range 1.6–14.8 D), and mean CDVA was 0.47 ± 0.2 logMAR (range 0.3–1.0 logMAR) [Snellen equivalent 20/50]. Manual dissection was performed in all cases. None of the examined eyes were converted to PK. An improvement in both topographic astigmatism (2.8 ± 0.9 D, *p* = 0.0135) and CDVA (0.23 ± 0.2 LogMAR, *p* = 0.0122) was recorded at 12-month follow-up. **Conclusions**: mDALK is a safe and effective surgical technique when applied to eyes previously treated with RK, with an observed improvement in CDVA and topographic astigmatism.

## 1. Introduction

Radial keratotomy (RK) is a refractive surgical procedure used to treat myopia. It consists of the creation of radial corneal incisions from the periphery to the paracentral optical zone to flatten the corneal curvature. Sato et al. first described the RK procedure in 1953 [1]. Over time, several complications of RK have been reported, including corneal perforation, decentered treatment, over- or under-correction, postoperative irregular astigmatism, and central corneal scarring [2,3]. Hence, this procedure has been largely replaced by laser refractive surgery. Rapid advances in excimer laser technology and surgical techniques, including the introduction of the femtosecond laser, have enabled refractive surgeons to treat a wider range of patients. These refinements are reflected in the improved refractive and visual outcomes and better safety profiles [2,3]. Although RK is no longer performed, presently there is a consistent number of patients suffering from previous RK-related visual disturbances. In such cases, visual recovery may be challenging, especially when rigid contact lenses are not well tolerated. Laser in situ keratomileusis [4,5] and photorefractive keratectomy [6] have been shown to be variably effective in managing post-RK refractive error and can expose patients to further complications, such as stromal haze and flap splitting [7]. Conversely, corneal transplantation appears to be a reasonable option to restore visual acuity in these cases, particularly in the presence of irregular astigmatism or opacity involving the visual axis. Corneal transplantation surgery has dramatically evolved over the last decades, with the standardization of partial thickness techniques that have boosted the short- and long-term safety outcomes. Deep anterior lamellar keratoplasty (DALK) represents the surgical technique of choice for corneal pathologies not involving the complex Descemet’s membrane (DM)-endothelium.

In fact, it has been demonstrated that corrected distance visual acuity (CDVA) measured after DALK is comparable to penetrating keratoplasty (PK); however, a lower risk of rejection and a lower endothelial cell loss have been documented in eyes treated with DALK compared to PK, providing a possible advantage in terms of graft survival [8,9,10]. Currently, DALK is most frequently performed with either of two techniques: by cleavage obtained with injection of air in the deep corneal stroma (“big-bubble technique”) [11] or by manual lamellar dissection (mDALK) [12,13]. Several authors have shown that there is no significant difference in terms of CDVA when comparing mDALK with “big-bubble” DALK [12,14].

We identified two case series by Pellegrini et al. and Einan-Lifshitz et al. investigating the safety and efficacy of the “big-bubble” DALK in previous RK and one series describing the outcome of the tuck-in DALK in the management of post RK keratectasia. In the first report, the success rate of big bubble formation was 87.5%, while in the second it appeared to be poorly predictable and 30% of surgeries had to be converted to PK. In fact, in the context of RK, DALK presents technical difficulties due to the presence of scars and/or possible pre-existing DM microperforations caused by the previous radial cut. In the third study, the authors reported satisfactory outcomes in terms of both visual recovery and postoperative astigmatism without significant complications [15,16,17].

The purpose of this study was to report the clinical outcomes and surgical complications of eyes previously treated with RK and undergoing modified manual DALK dissection.

## 2. Material and Methods

This is a non-comparative, interventional case series. We retrospectively reviewed the clinical charts of patients previously treated with RK and undergoing mDALK procedure at San Giovanni Addolorata Hospital (Rome, Italy); Mount Saint Joseph Hospital (Vancouver, Canada), and Tor Vergata University Hospital (Rome, Italy). The tenets of the Declaration of Helsinki were followed with informed consent for surgery as part of routine clinical care.

From 2017 to 2021, we identified thirteen eyes previously treated with RK and undergoing mDALK.

The indication for surgery was previous RK, with poor visual acuity due to irregular astigmatism or corneal scar and contact lens intolerance or contacts not being useful to improve the vision. The procedures were performed by three expert surgeons (AP, AI, and FA).

We collected data from preoperative and postoperative eye examinations including Snellen uncorrected distance visual acuity (UDVA), corrected distance visual acuity (CDVA), slit-lamp examination, tonometry, fundus examination, corneal topography (Pentacam, Oculus Optikgeräte GmbH, Wetzlar, Germany), and anterior segment-optical coherence tomography (AS-OCT) (Visante AS-OCT, Carl Zeiss Meditec, Germany and/or MS-39 AS-OCT, CSO, Florence, Italy).

### 2.1. Surgical Procedure

Surgeons performed a partial-thickness trephination with an adjustable suction trephine (Moria, Paris, France). The trephination never exceeded 80–100 µm from the thinnest AS-OCT pachymetry value at a variable diameter between 8.00 and 8.5 mm. The size of trephination was determined according to the horizontal corneal diameter. In all cases, donor and recipient cornea were trephined at the same diameter. A superficial keratectomy was performed after the trephination to expose a deep stromal plane. The surgeon hunted the most suitable previous keratotomy cut, defined as a vertical corneal incision reaching up to 90% of the entire corneal stroma according to the pre-operative anterior segment OCT evaluation when applicable. A careful manual dissection was performed, and a blunt spatula was used to recognize the deepest point of a keratotomy cut to perform a centripetal stromal manual dissection. The search for a desirable plane was carried out until the spatula could be inserted freely along the anatomical plane without any significant resistance. The aim was to reach the deep and regular stromal plane close to the pre-Descemetic layer. Then, the manual superficial keratectomy was performed in all quadrants.

For the donor graft, after removal of the corneal endothelium-DM complex from the donor cornea, the tissue was secured with a sixteen-bite 10-0 nylon running suture, taking care to avoid the peripheral radial cuts. At the end of the procedure, surgeons attempted to minimize astigmatism using intraoperative adjustment of continuous sutures.

Routine postoperative medications included Moxifloxacin 0.5% eye-drops four times daily for 1 week and a tapering regimen of topical steroid medication—typically Dexamethasone 0.1% every 1–2 h for 1 week, to be reduced over 8–12 months after surgery according to the surgeon preference.

All patients were observed at one day, one week, and one month after surgery, with a patient-tailored follow-up regimen dictated by clinical progress.

In line with Borderie et al., we defined graft failure as irreversible graft stromal oedema and corneal opacification. Similarly, graft rejection was defined in accordance with the site primarily affected by the reactive immunological process (i.e., epithelial and stromal) as any new corneal oedema and haze or sectoral stromal infiltration with or without subsequent neovascularization confined to the area of the allograft [16].

### 2.2. Statistical Analysis

**Figure 1:**
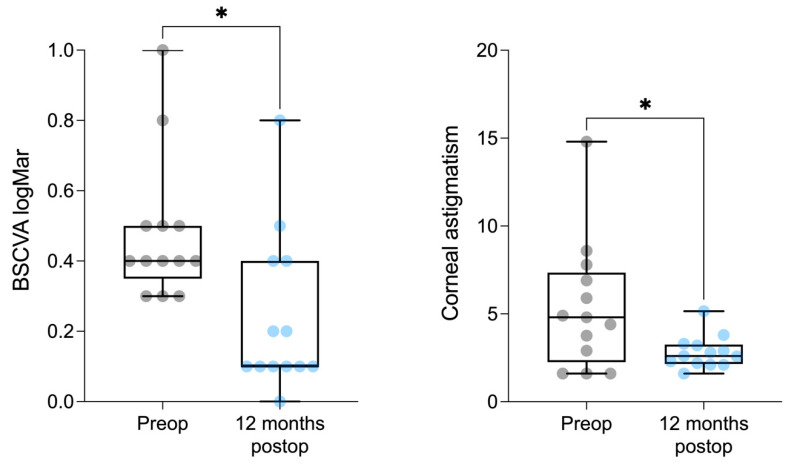
Boxplot showing the preoperative and postoperative CDVA and corneal astigmatism. * *p* < 0.05 Mann-Whitney two-tailed test. Boxplot shows medians, 25th and 75th percentiles as box limits, 10th and 90th as whiskers.

Data were recorded using Microsoft Excel (2021). Corrected distance visual acuity (CDVA) and corneal astigmatism were recorded preoperatively and postoperatively at month 3, month 6, month 9, and month 12; the results of CDVA were converted to logarithm of the minimum angle of resolution (logMAR) before statistical analysis. Statistical analysis was performed in GraphPad Prism (Version 9.5.0). Shapiro–Wilk tests were run to verify data normality, followed either by paired *t*-test (normally distributed data, mean topographic astigmatism before and 12 months after surgery) or Wilcoxon matched-paired signed rank test (not normally distributed data, CDVA before and 12 months after surgery) (Figure 1). Continuous variables were expressed as mean ± standard deviation (range). Statistical significance was defined as a *p*-value < 0.05.

## 3. Results

Thirteen eyes of eleven patients were included in the analysis (male 7/11, 63.6%). The mean age at the time of surgery was 48.2 ± 8.1 years (range 35–61 years). Indications for surgery were the presence of irregular astigmatism in 13 eyes (100%), stromal haze in 7 eyes (53.8%), and hyperopic shift in 7 eyes (53.8%). The average time between RK and transplant was 24.1 ± 5.9 years (range 10–30 years). All patients included were phakic. Eleven eyes were operated at San Giovanni Addolorata Hospital in Rome, Italy, one eye at Mount Saint Joseph Hospital in Vancouver, Canada, and one eye at Tor Vergata Hospital in Rome, Italy. Mean preoperative steepest keratometry (Kmax) was 53.4 ± 9.7 diopters (D) (range: 44.2–73.5 D), topographic astigmatism was 5.4 ± 3.7 D (range 1.6–14.8 D), and corneal thickness at the thinnest point was 451.2 ± 83.3 μm (range: 334–572 μm). The mean pre-operative CDVA was 0.47 ± 0.2 logMAR (range 0.3–1.0 logMAR). [Snellen equivalent 20/50]. Mean follow up was 37.4 months (Table 1).

**Figure 2:**
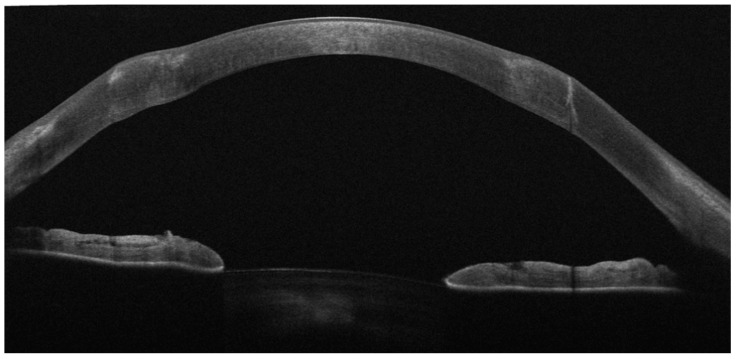
Postoperative AS-OCT. Thin residual stromal bed after mDALK in RK.

Manual dissection was performed in all cases. All patients underwent postoperative AS-OCT measurements to evaluate the residual host central stromal thickness. The mean residual central stromal thickness was 35.3 ± 4.7 μm (range 25–41 μm) (Figure 2).

None of the surgical procedures required conversion to PK. In one case only, we documented the formation of a double anterior chamber on postoperative day 1 due to a DM microperforation, successfully managed with a single slit-lamp intracameral air bubble injection. All patients had sutures removed between 8 and 10 months after the surgery. We analyzed the refractive and visual status at 12 months post-graft procedure.

**Figure 3:**
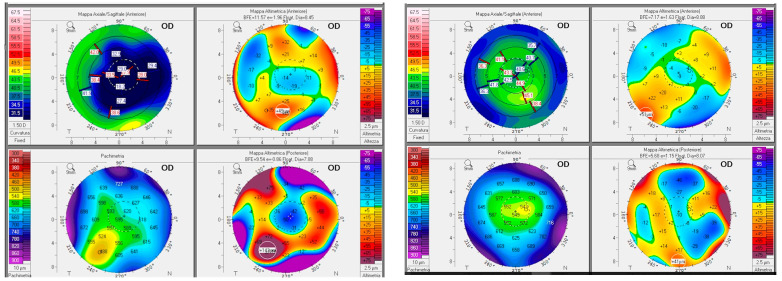
Preoperative (**left**) and postoperative (**right**) topography of patient 3.

**Figure 4:**
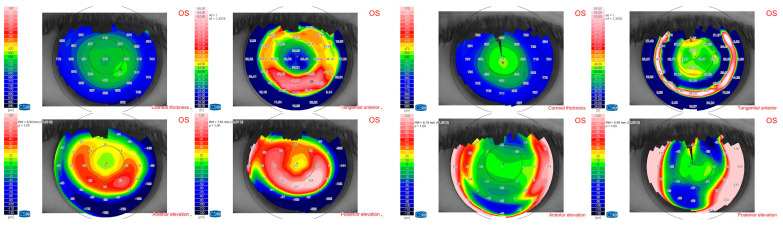
Preoperative (**left**) and postoperative (**right**) topography of patient 11.

Mean CDVA at the 12-month follow-up was 0.23 ± 0.2 logMAR (range from 0.0 to 0.8 logMAR) [Snellen equivalent 20/40], with a statistically significant improvement as compared to the preoperative CDVA (*p* = 0.0122). One patient only lost one line of CDVA because of cataract progression, and one patient lost three lines because of an epiretinal membrane (ERM) with foveoschisis development. At 12 months postoperatively, patients had a mean spherical equivalent of −6.8 ± 4.1 D (range from −0.5 to −13.5 D) and a mean topographic astigmatism of 2.8 ± 0.9 D (range from 1.6 to 5.15 D), showing a statistically significant improvement compared to the preoperative data (*p =* 0.0135) (Figure 3 and Figure 4).

No case of graft rejection and/or failure was observed during the follow-up period.

## 4. Discussion

In this case series, we report the outcomes of a modified mDALK dissection in post-RK eyes.

RK was a popular refractive surgery technique for correcting myopia in the 1980s and 1990s. The radial incisions made to flatten the cornea to reduce its refractive power can cause long-term visual complications and structural weakness of the cornea that may require surgical treatment.

Corneal ectasia, despite its rarity, can represent a long-term complication of RK. Early management is characterized by nonsurgical methods such as scleral contact lenses. However, advanced cases characterized by corneal haze, diurnal visual fluctuation, and irregular astigmatism in patients with contact lens intolerance require surgical intervention. Keratoplasty is the most frequently performed procedure, but it is challenging because it requires a large graft that includes the peripheral area of radial cuts. PK has been commonly employed in the past with different levels of effectiveness. Key considerations in full-thickness keratoplasty for post-RK ectasia involve the avoidance of radial incision detachment during trephination and the necessity of extra sutures.

Significant advancements in lamellar keratoplasty techniques have led to improvement in clinical outcomes. Endothelial cell loss rate is lower with DALK compared PK, allowing for improved graft longevity and survival rates [18]. Presently, DALK is the standard of care for treatment for corneal stromal diseases in the presence of both an intact DM and a healthy endothelium.

The big-bubble DALK technique relies on the splitting between the complex DM-endothelium and the corneal stroma with the help of forced air bubble injection, to allow separation between either the DM (type 2 bubble) or the pre-Descemetic layer (type 1 bubble) and the overlying stroma. Notably, the most common procedural complication observed in DALK surgery is DM tear, which might occur in up to 45.4% of eyes, with variable percentages depending on surgeons’ experience [12,19,20]. Nonetheless, as shown by Einan-Lifshitz et al., the success rate of big-bubble formation may plummet to about 10% when eyes previously treated with RK are considered [16].

In this context, the presence of corneal incisions, theoretically reaching up to 90% of the total corneal depth, might represent a major risk factor for big-bubble DALK conversion to PK. In fact, scar formation might extend to the pre-Descemetic layer and DM, thereby causing derangement of collagen fibrils and weakening of the overall structure. As a result, DM tears, micro-, or macroperforations might prevent a successful DALK. Furthermore, the air leakage through an RK cut can be commonly observed in post-RK eyes lowering the percentage of big-bubble formation.

As shown by our results, the mDALK technique may represent a more favorable surgical approach in RK cases. In fact, the direct visualization of the surgical plane as well as the controlled dissection of the corneal layers makes mDALK a safer procedure than big-bubble–DALK in the case of previous RK. In fact, as shown by our results, none of the eyes treated with mDALK needed an intraoperative PK conversion, while a double anterior chamber formation was only observed in one case (10%) that resolved after a single anterior chamber air injection. The low rate of complications is attributable to the surgeons’ experience. Once the posterior stromal plane is isolated, it is easier to avoid exposing the Descemet membrane, where microperforations might be present due to the previous cuts.

Postoperative CDVA observed in our study did not appear different than what has been reported by others [16,18,19,20,21]. Conversely, differences emerged when the refractive status reported in our study was compared with the results already reported in the literature, where the postoperative mean spherical equivalent was less myopic compared to our results [16,18,19,20,21]. This difference may be due to the selection criteria applied, as all the eyes included in our cohort were myopic before the RK procedure, with a presumably high axial length. In addition, as reported in the literature, DALK tends to produce a further myopic shift, determining a steepening of both K1 and K2 [22]. Furthermore, the analysis of visual acuity and mainly the refraction performed between 2 and 4 months post suture removal can be a bias since the refractive status can change up to 6 months after.

Limitations of our study were its retrospective nature and the small cohort size; nevertheless, it should be taken into consideration that this is the largest published series about the outcomes of DALK in RK.

As shown by our results, conducting a manual dissection from a pre-determined deep RK cut for DALK in post-RK eyes has proven safe and effective, with favorable conversion rate to PK and satisfactory visual outcomes, which appear comparable to the ones observed in the literature. Surgeons should be aware of the myopic shift tendency and counsel the patients accordingly to optimize the results. However, further studies on larger cohorts and longer observational periods are needed to further substantiate our findings.

## Figures and Tables

**Table 1:** Individual data of each patient.

Patient Number	Age at Time of DALK, Years	Gender	Time between RK and Keratoplasty, Years	CDVA (logMar) [Snellen Equivalent] Prior to Surgery	Pre-Operative Ks (Astigmatism), Diopters	Type of Keratoplasty	Lens Status	CDVA (logMar) [Snellen Equivalent] 12-Month Follow Up	Postoperative Ks 12-Month Follow Up (Astigmatism), Diopters
1	48	F	26	(0.4) [20/50]	K1 36.9 K2 38.6 (1.6)	Manual dissection DALK	Phakic	(0.2) [20/32]	K1 42.8 K2 45.6 (2.8)
2	57	M	20	(1) [20/200]	K1 45.6 K2 60.4 (14.8)	Manual dissection DALK	Phakic	(0.5) [20/63]	K1 42.4 K2 47.6 (5.15)
3	56	M	29	(0.4) [20/50]	K1 24.1 K2 29.0 (4.9)	Manual dissection DALK	Phakic	(0.4) [20/50]	K1 41.6 K2 43.2 (1.6)
4	35	M	10	(0.4) [20/50]	K1 34.6 K2 41.5 (6.9)	Manual dissection DALK	Phakic	(0.1) [20/25]	K1 44.0 K2 46.9 (2.9)
5	56	M	29	(0.3) [20/40]	K1 29.9 K2 35.9 (6)	Manual dissection DALK	Phakic	(0.4) [20/50]	K1 44.5 K2 47.7 (2.2)
6	61	M	30	(0.5) [20/63]	K1 29.4 K2 33.8 (4.4)	Manual dissection DALK	Phakic	(0.8) [20/125]	K1 41.8 K2 44.4 (2.6)
7	51	M	30	(0.3) [20/40]	K1 27.7 K2 35.5 (7.8)	Manual dissection DALK	Phakic	(0.2) [20/32]	K1 42.1 K2 45.4 (3.3)
8	41	F	16	(0.5) [20/63]	K1 37.6 K2 46.2 (8.6)	Manual dissection DALK	Phakic	(0.1) [20/25]	K1 43.8 K2 46.4 (2.6)
9	46	F	23	(0.4) [20/50]	K1 38.9 K2 41.8 (2.9)	Manual dissection DALK	Phakic	(0.1) [20/25]	K1 41.3 K2 43.5 (2.2)
10	48	M	25	(0.5) [20/63]	K1 41.4 K2 46.2 (4.8)	Manual dissection DALK	Phakic	(0.1) [20/25]	K1 43.5 K2 45.8 (2.3)
11	49	F	27	0.4 [20/50]	K1 33.06 K2 34.61 (1.6)	Manual dissection DALK	Phakic	(0.1) [20/25]	K1 40.8 K2 44.6 (3.8)
12	37	M	22	0.8 [20/125]	K1 43.3 K2 49.3 (6)	Manual dissection DALK	Phakic	(0.0) [20/20]	K1 44.2 K2 47.3 (2.1)
13	41	M	27	0.3 [20/40]	K1 44.4 K2 48.6 (3.8)	Manual dissection DALK	Phakic	(0.1) [20/25]	K1 45.3 K2 47.4 (2.1)

## Data Availability

The datasets generated during and/or analyzed during the current study are available from the corresponding author on reasonable request.

## Abbreviations

DALK: deep anterior lamellar keratoplasty; PK: penetrating keratoplasty; RK: radial keratotomy; LASIK: laser in situ keratomileusis; AS-OCT: anterior segment-optical coherence tomography.

## References

[1] Sato T., Akiyama K., Shibata H. (1953). A new surgical approach to myopia. Am. J. Ophthalmol..

[2] Hersh P.S., Kenyon K.R. (1990). Complications of radial keratotomy: Review of the literature and implications for a developing country. Indian J. Ophthalmol..

[3] Waring G.O., Lynn M.J., McDonnell P.J. (1994). Results of the prospective evaluation of radial keratotomy (PERK) study 10 years after surgery. Arch. Ophthalmol..

[4] Afshari N.A., Schirra F., Rapoza P.A., Talamo J.H., Ludwig K., Adelman R.A., Kenyon K.R. (2005). Laser in situ keratomileusis outcomes following radial keratotomy, astigmatic keratotomy, photorefractive keratectomy, and penetrating keratoplasty. J. Cataract. Refract. Surg..

[5] Sinha R., Sharma N., Ahuja R., Kumar C., Vajpayee R.B. (2011). Laser in-situ keratomileusis for refractive error following radial keratotomy. Indian J. Ophthalmol..

[6] Anbar R., Malta J.B., Barbosa J.B., Leoratti M.C., Beer S., Campos M. (2009). Photorefractive keratectomy with mitomycin-C for consecutive hyperopia after radial keratotomy. Cornea.

[7] Koch D.D., Liu J.F., Hyde L.L., Rock R.L., Emery J.M. (1989). Refractive complications of cataract surgery after radial keratotomy. Am. J. Ophthalmol..

[8] Reinhart W.J., Musch D.C., Jacobs D.S., Lee W.B., Kaufman S.C., Shtein R.M. (2011). Deep anterior lamellar keratoplasty as an alternative to penetrating keratoplasty a report by the american academy of ophthalmology. Ophthalmology.

[9] Maurino V., Aiello F. (2015). Glaucoma risks in advanced corneal surgery. Prog. Brain Res..

[10] Janiszewska-Bil D., Czarnota-Nowakowska B., Krysik K., Lyssek-Boroń A., Dobrowolski D., Grabarek B.O., Wylęgała E. (2021). Comparison of Long-Term Outcomes of the Lamellar and Penetrating Keratoplasty Approaches in Patients with Keratoconus. J. Clin. Med..

[11] Anwar M., Teichmann K.D. (2002). Big-bubble technique to bare Descemet’s membrane in anterior lamellar keratoplasty. J. Cataract. Refract. Surg..

[12] Knutsson K.A., Rama P., Paganoni G. (2015). Modified big-bubble technique compared to manual dissection deep anterior lamellar keratoplasty in the treatment of keratoconus. Acta Ophthalmol..

[13] Melles G.R., Lander F., Rietveld F.J., Remeijer L., Beekhuis W.H., Binder P.S. (1999). A new surgical technique for deep stromal, anterior lamellar keratoplasty. Br. J. Ophthalmol..

[14] Schiano-Lomoriello D., Colabelli-Gisoldi R.A., Nubile M., Oddone F., Ducoli G., Villani C.M., Mastropasqua L., Pocobelli A. (2014). Descemetic and predescemetic DALK in keratoconus patients: A clinical and confocal perspective study. Biomed. Res. Int..

[15] Pellegrini M., Yu A.C., Busin M. (2023). Large-Diameter Modified Big-Bubble Deep Anterior Lamellar Keratoplasty in Post-Radial Keratotomy Eyes. Arch. Ophthalmol..

[16] Einan-Lifshitz A., Belkin A., Sorkin N., Mednick Z., Boutin T., Kreimei M., Chan C.C., Rootman D.S. (2019). Evaluation of Big Bubble Technique for Deep Anterior Lamellar Keratoplasty in Patients With Radial Keratotomy. Cornea.

[17] Nagpal R., Sharma N., Bafna R.K., Muraleekrishna M., Vajpayee R.B. (2022). Tuck-in deep anterior lamellar keratoplasty for the management of post-radial keratotomy keratectasia. J. Cataract. Refract. Surg..

[18] Borderie V.M., Sandali O., Bullet J., Gaujoux T., Touzeau O., Laroche L. (2012). Long-term results of deep anterior lamellar versus penetrating keratoplasty. Ophthalmology.

[19] Gadhvi K.A., Romano V., Fernández-Vega Cueto L., Aiello F., Day A.C., Allan B.D. (2019). Deep Anterior Lamellar Keratoplasty for Keratoconus: Multisurgeon Results. Am. J. Ophthalmol..

[20] Gadhvi K.A., Romano V., Cueto L.F.-V., Aiello F., Day A.C., Gore D.M., Allan B.D. (2020). Femtosecond Laser-Assisted Deep Anterior Lamellar Keratoplasty for Keratoconus: Multi-surgeon Results. Am. J. Ophthalmol..

[21] Romano D., Aiello F., Parekh M., Levis H.J., Gadhvi K.A., Moramarco A., Viola P., Fontana L., Semeraro F., Romano V. (2023). Incidence and management of early postoperative complications in lamellar corneal transplantation. Graefe’s Arch. Clin. Exp. Ophthalmol..

[22] Fung S.S., Aiello F., Maurino V. (2016). Outcomes of femtosecond laser-assisted mushroom-configuration keratoplasty in advanced keratoconus. Eye.

